# Global Similarity Method Based on a Two-tier Random Walk for the Prediction of microRNA–Disease Association

**DOI:** 10.1038/s41598-018-24532-7

**Published:** 2018-04-24

**Authors:** Min Chen, Bo Liao, Zejun Li

**Affiliations:** 1 College of Information Science and Engineering, Hunan University, Changsha, 410082 China; 2 College of Computer Science and Technology, Hunan Institute of Technology, 421002 Hengyang, China

## Abstract

microRNAs (miRNAs) mutation and maladjustment are related to the occurrence and development of human diseases. Studies on disease-associated miRNA have contributed to disease diagnosis and treatment. To address the problems, such as low prediction accuracy and failure to predict the relationship between new miRNAs and diseases and so on, we design a Laplacian score of graphs to calculate the global similarity of networks and propose a Global Similarity method based on a Two-tier Random Walk for the prediction of miRNA–disease association (GSTRW) to reveal the correlation between miRNAs and diseases. This method is a global approach that can simultaneously predict the correlation between all diseases and miRNAs in the absence of negative samples. Experimental results reveal that this method is better than existing approaches in terms of overall prediction accuracy and ability to predict orphan diseases and novel miRNAs. A case study on GSTRW for breast cancer and conlon cancer is also conducted, and the majority of miRNA–disease association can be verified by our experiment. This study indicates that this method is feasible and effective.

## Introduction

MicroRNAs (miRNAs) refer to numerous evolutionarily conserved single-strand endogenous noncoded RNAs widely found in eukaryotes, and their length is equivalent to 20–25 nucleotides. miRNA accounts for 1% to 4% of the human genome^1–4^. In some cases, miRNAs participate in target gene regulation. They can identify and target an mRNA solution through base pairing after transcription, thereby controlling gene expression. An miRNA generally targets one or numerous mRNAs. MiRNA also plays an important role in many life processes, such as cell growth^5,6^, histological differentiation^7^, cell proliferation^8^, embryonic development^9^, apoptosis^10^ and metabolism^11,12^.

MiRNAs are closely related to cancers. For example, miR-21 and miR-223 are highly expressed in plasma samples of patients with gastric cancer compared with those in normal samples, and miR-218 has a significantly low expression^13^. The expression of miR-21 is closely associated with prostate cancer^14^, and this miRNA favors the replication of hepatitis B virus^15^. Toffanin *et al*.^16^ found that liver cancer has three subtypes, namely, proliferation correlation, interferon and Wnt signal. miR-517a performs a carcinogenic role in proliferation-related tumour subtypes and can promote the formation and development of subtype tumour. Different subtypes of breast cancer can be correctly classified by analysing miRNAs based on their expression spectrum^17^. Biotechnology involving locked nucleic acid is adopted to inhibit the miRNA activities of miR-21, miR-122 and miR-155 and effectively treat breast cancer in mice^18^. This technology has a clear targeting mechanism, so it is stable and weakly toxic. Thus, miRNAs may be useful for future clinical cancer treatment and drug design. The identification of disease-related miRNAs can also enhance studies on biomarker detection for the prognosis, diagnosis and treatment of complex human diseases.

Disease-associated miRNA mining methods based on biological experiments can accurately reveal disease-associated miRNAs. However, this method involves a long cycle and entails a high cost. In many methods, only one experiment can determine one disease-associated miRNA^19,20^. Thus, bioinformatics methods should be developed to identify disease-associated miRNAs quickly and accurately. In computational methods, computer technology based on existing biological experimental data is used to obtain multiple candidate miRNAs associated with a specific disease from a large number of miRNAs and thus provide reliable and comprehensive candidate miRNAs^19,21^. Given that an increasing number of disease-associated miRNA databases have been established^22–26^, computational methods can be effectively applied to predict the potential correlation between miRNA and diseases^27–33^ Bioinformatics prediction methods of miRNA–disease association can be generally divided into two categories: one is based on machine learning, and the other one is based on biological networks. In this study, the prediction method of miRNA–disease association is discussed on the basis of these two categories.

Many calculation methods based on the hypothesis that function-associated miRNAs are likely correlated with diseases exhibiting a similar phenotype have been proposed to predict the potential association between diseases and miRNAs^22,34,35^. In 2009, Jiang *et al*.^19^ developed a hypergeometric distribution calculation model to predict miRNA–disease association. They used the relationship between target genes to regulate miRNA and establish an miRNA similarity network. They also obtained special disease-associated miRNAs by using the human disease phenotype and miRNA function similarity. Li *et al*.^36^ introduced a method of gene function consistency to predict carcinogenic miRNA, used the functional consistency score of cancer-associated gene sets and miRNA target set to measure the similarity between diseases and miRNAs and showed the probability of the correlation between disease and miRNA. Xu *et al*.^37^ established a disease-associated miRNA prediction method that integrates the expression spectrum of miRNA and mRNA associated with a disease exhibiting phenotypic similarity. This method does not also rely on the known miRNA–disease association. With this method, the association probability between an miRNA and a disease is converted to the functional similarity between an miRNA target and a disease-associated gene for calculation. The known disease–gene association and the interaction with the miRNA target are used to calculate the correlation between miRNAs and diseases. This score is utilised to predict the ranking. However, these methods are highly dependent on the prediction of miRNA–target association. The false-positive result of target genes is high. As such, these methods cannot achieve a highly predictive performance. Rossi *et al*.^38^ designed a method named OMiR to predict the association between miRNA and diseases in OMIM. The overlapping degree of an miRNA locus with a disease gene locus in OMIM is calculated and used as miRNA–disease association. In this method, information regarding miRNA–disease association, miRNA target, pathogenesis and other aspects are unnecessary to determine miRNA–disease association. Pasquier *et al*.^39^ revealed the information on the disease-related miRNA through semantic distribution. According to a case study on breast cancer, this method can be applied to determine new miRNA–disease association and identify pseudocorrelation in the database.

Xuan *et al*.^40^ proposed a prediction method, namely, HDMP, based on its most similar k neighbours. The functional similarity of miRNA, the phenotypic similarity of disease, the semantic similarity of disease and the unknown association between miRNAs and diseases are used to establish a similar network and to predict the potential miRNA–disease association by using the k neighbours and miRNA functional similarity. With this method, only the information of the miRNA’s neighbour is considered in its ranking system, and a local similarity measure instead of a global measure is used. Thus, this method cannot be applied to some diseases without the known related miRNA. Many studies have shown that global network similarity can effectively improve prediction performance. In 2012, Chen *et al*.^41^ introduced a method named RWRMDA to predict miRNA–disease association based on global network similarity. They predicted a pathogenetic miRNA through a restarted random walk. Firstly, they integrated the miRNA–miRNA functional similarity and the known miRNA–disease associated information. Secondly, they initialised each miRNA as the probability of the starting node to execute a random walk algorithm in an integrated network until the algorithm is converged. A stable probability is obtained to rank the candidate miRNAs. Compared with the local similarity network, the global similarity network can improve the prediction accuracy. However, this method cannot predict new diseases without the known association. Chen *et al*.^42^ created a method, namely, Net-CBI, to predict the miRNA–disease association by considering the network conformance of disease. Chen *et al*.^43^ further calculated the global network similarity by determining the Laplacian score of graphs and proposed an miRNA–disease association prediction method based on random walk, namely, NetGS. However, too many parameters are present in these two methods. Gu *et al*.^44^ designed a network conformance method, which is called NCPMDA, to predict miRNA–disease association. This method is nonparametric, and it can simultaneously predict the miRNA–disease association among all diseases. No negative samples are needed in this method, and it can be applied to predict isolated diseases and new miRNAs.

Xuan *et al*.^45^ designed a computation model named MIDP based on random walk algorithm. This algorithm walks in a two-tier network composed of the disease similarity, miRNA similarity, and known miRNA–disease association. This model can predict diseases without the known association with miRNA. Liu *et al*.^46^ established a new prediction model by conducting a random walk algorithm on the heterogeneous networks of multisource data. Chen *et al*.^47^ also developed a new computation method named WBSMDA, which is mainly used to integrate the known miRNA–disease association, miRNA functional similarity, semantic disease similarity and Gaussian interaction profile kernel similarity of disease and miRNA. This method can predict new diseases without known associated miRNA, and it can predict any non-disease-associated MiRNA. However, the performance of WBSMDA is still unsatisfactory. Chen *et al*.^48^ established a heterogeneous graphics method named HGIMDA to predict miRNA–disease association. They also revealed the potential miRNA–disease association by establishing a heterogeneous graph composed of miRNA functional similarity, disease semantic similarity, Gaussian interaction profile kernel similarity and miRNA–disease association verified by experiments. You *et al*.^49^ also introduced a new path-based miRNA–disease association prediction method named PBMDA. This method can be used to predict new diseases without the known associated miRNA and the new miRNA without the known associated disease by integrating different types of heterogeneous biological data sets. This method can be used to prioritise unknown miRNA in all of the diseases. Chen *et al*.^50^ introduced a model based on the Super disease and Super miRNA to predict SDMMDA, which is the miRNA–disease association method. They integrated the known miRNA–disease association, disease semantic similarity, miRNA functional similarity and Gaussian interaction profile kernel similarity SDMMDA can be used to predict new diseases and miRNAs without any known association.

However, the disease-associated miRNAs verified by these experiments are insufficient. The comprehensive consideration of protein, target gene and other biological information can help predict miRNA–disease association. In 2013, Shi *et al*.^51^ used a miRNA–disease associated computation model. They established a complex network by integrating miRNA–target interactions, disease–gene associations and PPI. They used a random walk algorithm for prediction. Mork *et al*.^52^ also proposed a method called miRPD. This model integrates the protein–disease association and miRNA–protein interaction to further predict new miRNA–disease association. With this method, disease-associated miRNAs can be analysed, and disease-associated proteins can be predicted. Shi *et al*.^53^ proposed a method to integrate various types of genome data and predict miRNA–disease association, CHNmiRD. They also identified miRNA–disease association by integrating protein–protein data, gene noumenon data, experimentally verified miRNA–target data, phenotypic information of disease, the known miRNA–disease association, and other genome and phenotypic data.

Machine learning-based methods have been widely used in bioinformatics research^54–56^, including predicting miRNA–disease association. In 2010, Jiang *et al*.^57^ introduced a new method based on genomic data integration. A naive Bayesian model is used to integrate substantial data resources and to establish a functional prediction model among genes. Jiang *et al*.^58^ also proposed positive sample data from negative sample data by using a support vector machine. With this method, features are extracted from miRNA–target and phenotypic similarity data. Xu *et al*.^59^ proposed a method involving an miRNA target topology disorder network, which is used to predict prostatic cancer-associated miRNAs by using prostatic cancer as an example. Qabaja *et al*.^60^ also proposed a protein network based on a Lasso regression model to excavate the miRNA–disease association. Lasso regression model is utilised to identify disease-associated miRNAs. Zeng *et al*.^61^ also predicted the association between miRNAs and diseases by using two kinds of multipath methods. Unfortunately, these machine-based learning methods require known disease-associated miRNA-negative sample information. Thus, negative miRNA–disease association information is difficult to obtain. In 2014, Chen *et al*.^62^ introduced a semi-supervised algorithm based on a regularised least square method (RLSMDA) to predict potential miRNA–disease association. This method is used to predict potential miRNA–disease association based on a semi-supervised learning framework. No negative miRNA–disease-related information is needed in this method. Thus, RLSMDA can be used to predict a disease without any known associated miRNA. Chen and Huang^63^ proposed a computational model named LRSSLMDA,based on Laplacian Regularized Sparse Subspace Learning. The model integrated statistical feature profile of miRNAs and diseases and graph theoretical feature profile into a common subspace. Experimental results showed that the proposed method outperformed ten previous models and indicated the model’s superior performance. Chen *et al*.^64^ developed an miRNA-disease association prediction appoach called EGBMMDA by integrating Extreme Gradient Boosting Machine with miRNA functional similarity, disease semantic similarity, and known miRNA–disease associations into a unified framework. The framework was the first decision tree learning-based method to predict miRNA–disease associations.

Against miRNA similarity data deficiency, scarcely known relationship between miRNAs and diseases, and almost no negative sample, based on miRNA-miRNA network and disease-disease network, Zeng *et al*.^65^ proposed a method for predicting miRNA–disease association by suing a matrix completion algorithm. This method provides a new method to solve deficiency in miRNA–disease association data. This method can also be used to predict new diseases and pathogenic miRNAs. Peng *et al*.^66^ predicted miRNA–disease association by using an improved low-rank matrix recovery algorithm. Li *et al*.^67^ also introduced a method (MCMDA) to predict miRNA–disease association by using a matrix completion algorithm. Compared with previous methods, this algorithm is effective in low-level miRNA–disease matrix completion.

In 2014, Li *et al*.^68^ developed a toxicology framework of computation system by using the recommendation system. This framework can predict new associations among environmental factors, miRNAs and diseases by integrating the structural similarity of environmental factors and phenotypic similarity of disease. Considering social network analysis, Zou *et al*.^69^ introduced a method to predict miRNA–disease association based on social network analysis. They used two kinds of social network analysis methods, namely, KATZ and CATAPULT, to analyse a heterogeneous network. However, the disadvantage that there are only positive and unmarked samples in miRNA–disease association are overcame, Chen *et al*.^70^ also designed a new K-nearest neighbour algorithm (KNN)-based disease association sorting algorithm named RKNNMDA and integrated the functional similarity of miRNA, semantic similarity of disease, Gauss’s nuclear spectrum interactions and known miRNA–disease association. KNN is used to search the KNN of miRNAs and diseases and resorted K nearest neighbours based on the SVM sorting model. Chen *et al*.^71^ also introduced a method named restricted Boltzmann machine (RBM), which is used to predict different types of miRNA–disease association, including RBMMMDA. RBMMMDA can predict miRNA–disease association and obtain this associated type. However, the parameters of this method are difficult to know.

In summary, these methods have various limitations in predicting miRNA–disease association. Firstly, some methods strongly depend on incomplete and incorrect data sets, such as miRNA–target methods. Secondly, some machine learning methods require negative samples. However, these negative samples are difficult to obtain. Thirdly, some methods do not use information regarding the miRNA family or cluster. Finally, some methods cannot be applied to predict the isolated diseases and new miRNAs. Therefore, new methods should be developed and modified. In this study, a hypothesis is examined. This hypothesis states that the global network similarity measure is more suitable to identify the association between diseases and miRNAs than the local network similarity measure. The main contributions of this paper are as follows:

1. Global network similarity, fully used disease network and miRNA network information.
2. No negative sample is needed.
3. The miRNA family information and various biological data are integrated to capture new potential association information.
4. This method can be used to predict the isolated disease and new miRNA with good cross validation performance.

## Results

### Parameter selection and performance evaluation

To validate the prediction performance of the proposed algorithm, we tested the gold benchmark data set and validate its performance by using leave-one-out cross validation. The specific process is as follows: a known miRNA–disease relation pair is used as the test sample in each experiment, and other relation pairs are used as training samples; after the model training is completed, all known relation pairs are used as the testing sample to test once to predict the testing sample; To evaluate the leave-one-out cross validation result, we use the ROC curve, AUC and other indices. For the ROC curve, the true positive rate is set as the ordinate, and the false positive rate is utilised as the abscissa. After numerous pairs of the true positive and false positive rates are obtained by changing the threshold, the ROC curve is obtained through plotting. The AUC value is the area under the ROC curve. If the ROC curve is closer to the upper left corner, the area under the curve is large, and the prediction performance is enhanced.

The method proposed in this study mainly involves four parameter categories, namely, the restart parameters γ and *θ* for the restarted random walk algorithm, equilibrium parameters α and β for Laplacian score of graphs, disease and miRNA seed initialization weight parameters λ and η, and miRNA space weight parameter *w*. The selection of the four categories of parameters and their influences are discussed in this study.

**Figure 1:**
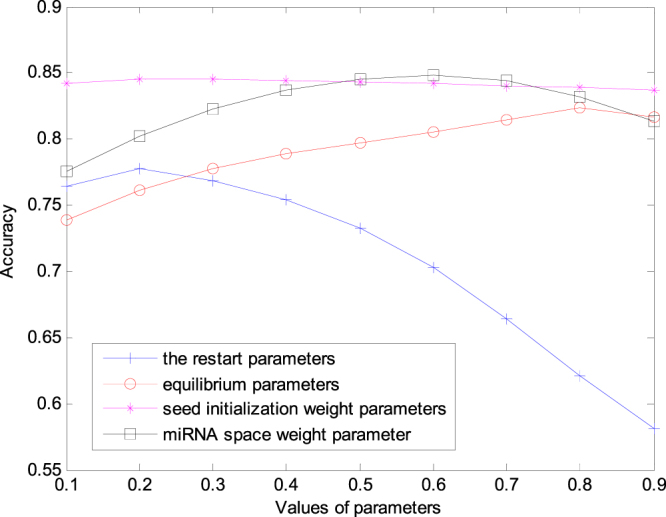
The effect of parameters on the GSTRW performance.

In the restarted random walk algorithm, γ and *θ* refer to the probability that random walk is conducted again after randomly backing to the source node. If γ and *θ* are high, the probability of going back to the node for each step is higher. For simplicity, γ and *θ* are set to be the same. To validate the effects of γ and *θ* on the performance of prediction algorithm, we fix the other parameters (α = β = 0.3, λ = η = 0.9, *w* = 0.5) and change γ and *θ*. In this process, 0.1 is set as a step length, and 0.1 is changed to 0.9 to cross validate and calculate the AUC value. The experimental result is shown in Fig. 1. As shown in Fig. 1, when γ and *θ* increase from 0.1 to 0.2, the AUC value increases. Using the maximum value, we obtain the best prediction performance. When γ and *θ* increase from 0.2 to 0.9, the AUC value decreases slowly.

The equilibrium parameter α for the Laplacian score of graphs in the miRNA network and the equilibrium parameter β for the Laplacian score of graphs are the same. To validate the effects of these parameters on the performance of the prediction algorithm, we firstly fix the other parameters (γ = *θ* = 0.2, λ = η = 0.9, *w* = 0.5), and we change the α and β values by considering 0.1 as a step length, and 0.1 is changed to 0.9. As shown in Fig. 1, the AUC value increases slowly as α and β increase. When α = β = 0.8, the maximum AUC is achieved, with a good prediction performance.

To predict the isolated disease and new miRNA and to improve the prediction accuracy, we initialise the disease and miRNA seeds. The initialisations of the weight parameters λ and η determine the contributions of other diseases and miRNAs to the initial vector. To validate its influence on the performance of the algorithm, we fix the values of the other parameters (γ = *θ* = 0.2, α = β = 0.8, *w* = 0.5) and change λ and η (starting from 0 to 0.9) for cross validation. As shown in Fig. 1, the AUC value is the highest, and λ and η are 0.2. With the increase in λ and η, it is slightly reduced; however, this reduction is not evident.

The similarity information on miRNAs and diseases should be fully used to obtain the best prediction performance. Using the two-tier random walk algorithm, we use the walk of the disease seed in the miRNA network to obtain a stable vector. The Pearson coefficients of this stable vector and miRNA global similarity are calculated as the prediction score of the disease in the miRNA global similarity network. The walk of miRNA seed in the disease network is utilised to determine a stable vector, and the Pearson coefficient of this stable vector and disease global similarity is calculated as the miRNA prediction score in the disease global similarity network. Finally, these two scores are weighted to obtain the final miRNA–disease association score. The miRNA network weight parameter is set to be $w(0\le w\le 1)$, and 1−w is the weight of the disease network. When *w* is greater, the weight of the miRNA network is higher. It indicates that, we hope the prediction result will consider more miRNA information. At this moment, the miRNA-based functional similarity plays a key role in the prediction of disease-associated miRNA. If *w* is smaller, then the prediction result more considers the prediction result of the disease-related information.

According to the previous discussion, the values of the other parameters are fixed (γ = *θ* = 0.2, α = β = 0.8, λ = η = 0.2), and *w* is changed from 0 to 0.9. When *w* is increased from 0.1 to 0.6, AUC gradually increases. When *w* is increased from 0.6 to 0.9, AUC gradually decreases. When *w* is 0.4, the prediction result is the best. These results indicate that our prediction results are dependent on the miRNA similarity.

**Figure 2:**
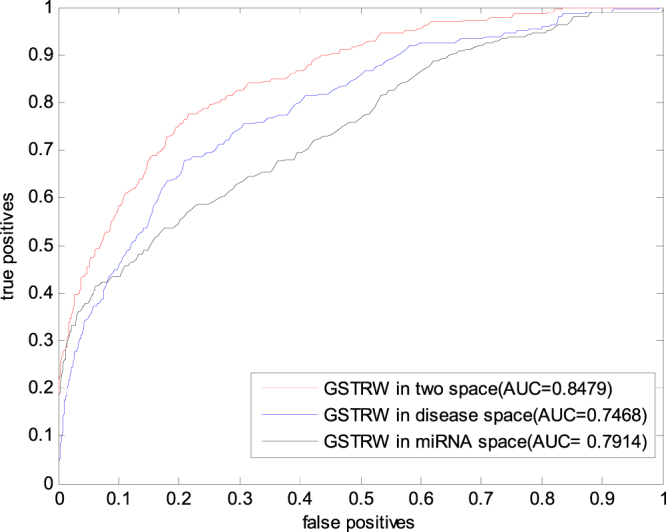
ROC curves and AUC values of GSTRW based on LOOCV in different situations.

Our proposed method not only makes use of diseased seeds to walk in the miRNA network, but also utilizes the miRNA seeds to walk in the diseased network. In order to illustrate the superiority of our method, we analyze the following situations in the experiment: 1) Prediction performance in miRNA networks and disease bi-level networks; 2) Prediction performance in miRNA networks only; 3) Prediction performance of walking in disease networks. Using a cross validation in the gold benchmark dataset validation, the experimental results shown in Fig. 2.

Obviously, GSTRW showed satisfactory predictive performance with a AUC value of 0.8479, whereas AUC was only 0.7914 in the miRNA network and 0.7468 in the diseased network, mainly due to GSTRW not only walking in the miRNA global similarity network but also walking in the global similarity network of the disease, the global similarity between the miRNA and the disease is taken into full consideration. Only walking in a single network only considers the global similarity of the miRNA or the disease.

### Comparison with other methods

So far as we know, there are some methods with better prediction performance of miRNA-disease association, including HDMP^40^, RLSMDA^62^, NetCBI^42^ and an algorithm based on network global information proposed by Shi *et al*.^51^. HDMP cannot be used to predict the relationship between isolated diseases and miRNAs. Thus, no other method can be compared with the method proposed in this paper.The method developed by shi *et al*.^51^ integrated the information of disease gene associations, miRNA target interactions, and protein interactions which were totally different from the information used in this paper, so the method predicted by Shi *et al*. cannot be fairly compared with GSTRW. The information used by RLSMDA and NetCBI is similar to that discussed in this study. Moreover, these three methods can be used to predict the isolated miRNA–disease association. Therefore, we compare these three methods in the present study.

**Figure 3:**
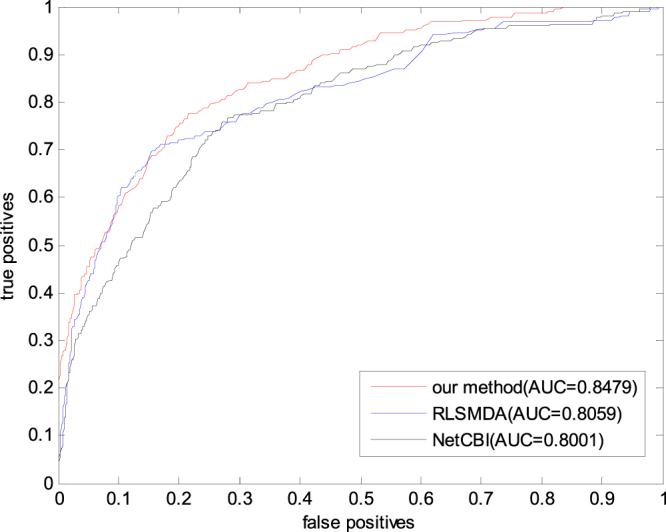
The ROC curves and AUC values of RLSMDA, NetCBI and our method(GSTRW).

On the basis of the previous section, we set the parameters as follows: γ = *θ* = 0.2, α = β = 0.8, λ = η = 0.2, *w* = 0.6. The experimental result is shown as Fig. 3. As shown in Fig. 2, the method proposed in this paper is better than RLSMDA and NetCBI in terms of the prediction performance.

The AUC values obtained from the experiments by RLSMDA and NetCBI are different from the given value in the original paper,The main reason for this difference is that the data sets adopted are different. This difference is attributed to the following: in the data set adopted by RLSMDA in the original paper, each miRNA is related to an average of 5.147 diseases, and each kind of disease is associated with an average of 10.18 miRNAs. However, the gold benchmark data set is adopted in this paper, and each miRNA is related to an average of 2.27 diseases. Each kind of diseases is associated with an average of 4.41 miRNAs. Thus, the available known information in the present study is much less than that in the original. Therefore, the prediction results are different. NetCBI adopts the same data set as we have used in this paper. However, redundancy removal is not performed in NetCBI, so the available known information in this paper is reduced, and the corresponding prediction result is changed. Therefore, this method exhibits good performance in the prediction of miRNA–disease association.

To validate the insensitivity of the proposed method to the data set in this paper, we carried out the comparative experiment on the predictive dataset. The experimental method is also leave-one-out cross validation.

**Figure 4:**
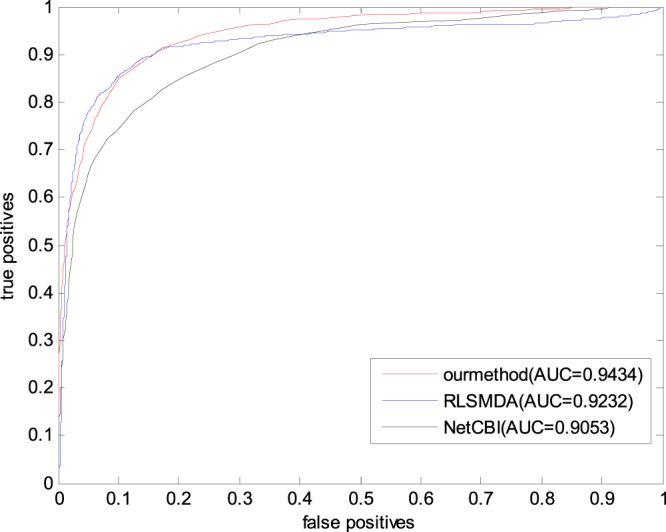
The prediction results of RLSMDA, NetCBI and GSTRW on the predictive dataset.

The experimental result is shown in Fig. 4. As shown in Fig. 4, the prediction accuracy of several methods is slightly improved. This phenomenon is attributed to the following: the known miRNA–disease information is increased more than the benchmark data set information in the predictive dataset. However, the available known information likely increases. Moreover, the prediction performance of GSTRW is better than those of the two other methods in this data set.

**Figure 5:**
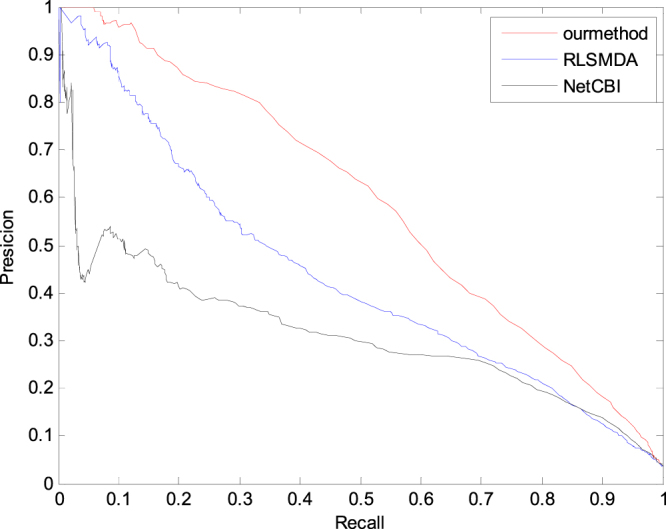
The Precision-recall curves of RLSMDA, NetCBI and GSTRW.

The accuracy, recall rate and accuracy–recall curve are also common indices. In this paper, on the basis of this standard, we adopt leave-one-out cross validation to compare RLSMDA, NetCBI and GSTRW. In Fig. 5, GSTRW is better than the existing method.

**Figure 6:**
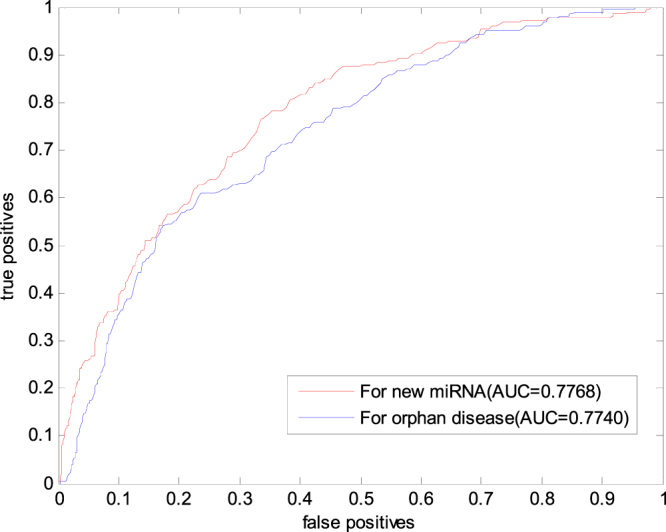
The ROC curve and AUC values of NetGS for new miRNA and orphan disease.

Orphan disease refers to a type of diseases with completely unknown miRNA-associated information. We simulate the isolated disease by removing the known relationship between the disease to be inquired and all miRNAs. To predict by using the proposed method in this paper, we use each disease as a test sample. The leave-one-out cross validation is adopted to test the gold data set. The prediction result is evaluated by the ROC curve and the AUC value. The prediction result is shown in Fig. 6. The AUC value is 0.7740, indicating that the proposed method elicits a certain effect on the prediction of the relationship between the isolated disease and miRNA.

In the recent years, an increasing number of miRNAs have been found, but their relationship with diseases is mostly unknown. This problem poses a challenge to the prediction algorithm. At present, many prediction methods cannot solve these problems. To validate the effectiveness of the proposed method in this paper in predicting the new miRNA–disease association, we remove the predicted association between miRNAs and all diseases. The proposed method is used to predict the removed association information. In addition, the leave-one-out cross validation is adopted to verify the gold benchmark data set. The AUC value reaches 0.7768, indicating that the proposed method has good performance for the prediction of the association between new miRNAs and diseases.

### Case study

According to the previous section, the proposed method in this paper has good prediction performance. On the basis of the predicted data set, we conduct a case study on breast cancer and liver cancer to evaluate the independent predictive ability of GSTRW.

Firstly, the GSTRW method is adopted to predict these two diseases. Afterwards, the prediction result is searched in the update of HMDD, miR2disease and dbDEMC datasets and other data sets to determine whether it is found or not. Tables 1 and 2 show the top 50 miRNAs associated with breast cancer and colon cancer that are predicted by our method, respectively.

**Table 1:** The top 50 breast cancer-related miRNAs candidates predicted by GSTRW and the confirmation of these associations.

Rank	miRNA name	evidences	Rank	miRNA name	evidences
1	hsa-mir-16	HMDD, dbDEMC	26	hsa-mir-32	dbDEMC
2	hsa-let-7i	HMDD,mir2disease,dbDEMC	27	hsa-mir-196b	dbDEMC
3	hsa-let-7b	HMDD, dbDEMC	28	hsa-mir-130a	dbDEMC
4	hsa-let-7e	HMDD,dbDEMC	29	hsa-mir-98	dbDEMC, miR2disease
5	hsa-let-7c	HMDD,dbDEMC	30	hsa-mir-199b	HMDD,dbDEMC
6	hsa-let-7g	HMDD,dbDEMC	31	hsa-mir-335	HMDD,mir2disease,dbDEMC
7	hsa-mir-373	HMDD,mir2disease,dbDEMC	32	hsa-mir-137	HMDD,dbDEMC
8	hsa-mir-92a	HMDD	33	hsa-mir-224	HMDD,dbDEMC
9	hsa-mir-92b	dbDEMC	34	hsa-mir-192	dbDEMC,
0	hsa-mir-223	HMDD, dbDEMC	35	hsa-mir-182	HMDD,mir2disease,dbDEMC
11	hsa-mir-126	HMDD,mir2disease,dbDEMC	36	hsa-mir-27a	HMDD,mir2disease,dbDEMC
12	hsa-mir-101	HMDD,mir2disease,dbDEMC	37	hsa-mir-150	HMDD, dbDEMC
13	hsa-mir-191	HMDD,mir2disease,dbDEMC	38	hsa-mir-124	HMDD,mir2disease,dbDEMC
14	hsa-mir-29c	HMDD,mir2disease,dbDEMC	39	hsa-mir-95	dbDEMC
15	hsa-mir-18b	HMDD,dbDEMC	40	hsa-mir-532	Unconfirmed
16	hsa-mir-372	dbDEMC	41	hsa-mir-520b	HMDD,dbDEMC
17	hsa-mir-181a	HMDD,mir2disease,dbDEMC	42	hsa-mir-491	Unconfirmed
18	hsa-mir-203	HMDD,mir2disease,dbDEMC	43	hsa-mir-183	HMDD,dbDEMC
19	hsa-mir-106a	dbDEMC	44	hsa-mir-142	Unconfirmed
20	hsa-mir-99b	dbDEMC	45	hsa-mir-135a	HMDD
21	hsa-mir-15b	dbDEMC	46	hsa-mir-22	HMDD,dbDEMC
22	hsa-mir-128b	miR2Disease	47	hsa-mir-23b	HMDD,dbDEMC
23	hsa-mir-30e	Unconfirmed	48	hsa-mir-449a	dbDEMC
24	hsa-mir-24	HMDD,dbDEMC	49	hsa-mir-449b	dbDEMC
25	hsa-mir-100	HMDD,dbDEMC	50	hsa-mir-31	HMDD,mir2disease,dbDEMC

Forty-six of the top 50 potential breast cancer miRNAs candidates have been confirmed based on the update HMDD, dbDEMC and mir2disease.

**Table 2:** The top 50 colon cancer-related miRNAs candidates predicted by GSTRW and the confirmation of these associations.

Rank	miRNA name	evidences	Rank	miRNA name	evidences
1	hsa-mir-125b	dbDEMC	26	hsa-mir-429	dbDEMC
2	hsa-mir-16	HMDD,dbDEMC	27	hsa-mir-203	dbDEMC,miR2Disease
3	hsa-mir-15a	HMDD,dbDEMC	28	hsa-mir-106b	HMDD,mir2disease,dbDEMC
4	hsa-mir-222	dbDEMC	29	hsa-mir-194	dbDEMC,miR2Disease
5	hsa-mir-199a	Unconfirmed	30	hsa-mir-196a	dbDEMC,miR2Disease
6	hsa-mir-181b	dbDEMC,miR2Disease	31	hsa-mir-302b	HMDD,dbDEMC
7	hsa-mir-25	dbDEMC,miR2Disease	32	hsa-mir-15b	dbDEMC,miR2Disease
8	hsa-mir-92b	Unconfirmed	33	hsa-mir-372	dbDEMC,miR2Disease
9	hsa-mir-9	dbDEMC	34	hsa-mir-181a	dbDEMC,miR2Disease
10	hsa-mir-29a	HMDD,mir2disease,dbDEMC	35	hsa-mir-224	dbDEMC,miR2Disease
11	hsa-mir-451	dbDEMC,miR2Disease	36	hsa-mir-219	Unconfirmed
12	hsa-mir-200a	Unconfirmed	37	hsa-mir-183	dbDEMC,miR2Disease
13	hsa-mir-34c	miR2Disease	38	hsa-mir-30d	dbDEMC
14	hsa-mir-146a	HMDD,dbDEMC	39	hsa-mir-218	dbDEMC
15	hsa-mir-18b	dbDEMC	40	hsa-mir-137	HMDD,mir2disease,dbDEMC
16	hsa-mir-135b	HMDD,mir2disease,dbDEMC	41	hsa-mir-30b	dbDEMC
17	hsa-mir-205	HMDD,dbDEMC	42	hsa-mir-339	miR2Disease
18	hsa-mir-29c	dbDEMC	43	hsa-mir-151	dbDEMC
19	hsa-mir-373	Unconfirmed	44	hsa-mir-30e	dbDEMC
20	hsa-mir-146b	dbDEMC	45	hsa-mir-10a	dbDEMC,miR2Disease
21	hsa-mir-214	dbDEMC	46	hsa-mir-31	dbDEMC,miR2Disease
22	hsa-mir-34b	dbDEMC,miR2Disease	47	hsa-mir-103	Unconfirmed
23	hsa-mir-20b	dbDEMC	48	hsa-mir-153	Unconfirmed
24	hsa-mir-93	dbDEMC,miR2Disease	49	hsa-mir-95	dbDEMC,miR2Disease
25	hsa-mir-125a	dbDEMC,miR2Disease	50	hsa-mir-302d	Unconfirmed

Forty- two of the top 50 potential conlon cancer miRNAs candidates have been confirmed based on the update HMDD, dbDEMC and mir2disease.

Breast cancer is a major fatal disease that threatens the life and health of women at present. Breast cancer-associated miRNAs should be identified to further understand the pathogenesis, treatment and prognosis of breast cancer.

In the prediction data set, 78 miRNAs are associated with breast cancer. As shown in Table 2, among the top 50 miRNAs associated with breast cancer predicted by GSTRW, 46 are verified by the three databases. The first 20 associations were all confirmed and only 2 of the first 40 MiRNAs were unconfirmed which are hsa-mir-30e ranked 23rd and hsa-mir-532 ranked 40th. However, Lin *et al*.^72^ demonstrated that hsa-mir-30e is down-regulated in breast cancer tissues. Ben-Hamo *et al*.^73^ found that breast cancer patients target the GATA3 pathway via hsa-miR-532 whereas GATA3 regulates hormone-sensitive breast cancer phenotype. The third key factor, hsa-mir-491, was not identified, but Shi *et al*.^74^ found that hsa-mir-491 is down-regulated in gastric cancer patients and has an inhibitory effect on cell proliferation. The fourth unproven has-mir- 142, Isobe *et al*.^75^ found that miR-142 regulates the tumorigenicity of human breast cancer stem cells via the WNT signaling pathway.This result indicates that the proposed method in this paper has a good practical value.

Colon cancer has a high malignant degree, and it develops rapidly without any symptoms in an early stage. If a certain explanation can be given on the basis of molecular perspectives, then it surely helps diagnose and treat diseases. Thus, colon cancer-associated miRNA should be identified.

In the prediction data set, 37 miRNAs are associated with the occurrence and development of lcolon cancer. GSTRW is used to sort miRNAs that are unknown to associate with colon cancer.

GSTRW finds colon cancer-associated miRNAs in which 42 miRNAs can be found in updated data sets such as HMDD, miR2disease and dbDEMC(Table 2). The first unverified miRNA is hsa-mir-199a ranked 5 and the second is hsa-mir-92b ranked 8 and hsa-mir-200a ranked 12 and hsa-mir-373 ranked 19. However, for these unverified miRNAs in the above three databases, some supportive evidence was obtained by searching the relevant literature. Nonaka *et al*.^76^ found that miR-199a can be used as a serum biomarker for colorectal cancer. Mussnich *et al*.^77^ found that miR-199a and miR-375 affect the sensitivity of colon cancer cells to cetuximab by targeting PHLPP1. Niu *et al*.^78^ believe that hsa-miR-92b can be used as circulating microRNA in colorectal cancer reference gene. Pichler *et al*.^79^ found that Mir-200a affects the prognosis of patients with rectal cancer by regulating the expression of genes involved in stromal metastasis of epithelial cells. Tanaka *et al*.^80^ found that the apparent silencing of microRNA-373 plays an important regulatory role in colon cancer cell proliferation.

### Applicability of GSTRW to predict orphan diseases

In order to verify the ability of GSTRW to predict the orphan diseases, we deleted the known association of miRNAs associated with validated diseases, which ensures that we only use the similarity information of validated and other diseases as well as those associated with other diseases information. We used breast and colon cancer as a case study and the results are shown in Tables 3 and 4, respectively. For breast cancer, we removed the association of 78 known breast cancers with miRNAs and predicted the association of potential miRNAs with breast cancer using GSTRW. Of the top 50 predicted miRNAs, 49 were found in the HMDD, miR2disease, and dbDEMC databases can be found. The only one unverified by database was the 46th ranked hsa-mir-184. Yang *et al*.^81^ used immunohistochemical methods to study breast tumor subtypes and found that there is expression differences on hsa-miR-365, hsa-miR-1238 and hsa-miR-184.

**Table 3:** The top 50 breast cancer-related miRNAs candidates predicted by GSTRW with removed all known breast cancer-miRNA associations and the confirmation of these associations.

Rank	miRNA name	evidences	Rank	miRNA name	evidences
1	hsa-mir-21	HMDD,mir2disease,dbDEMC	26	hsa-mir-10a	HMDD,mir2disease,dbDEMC
2	hsa-mir-146a	HMDD,mir2disease,dbDEMC	27	hsa-mir-141	HMDD,mir2disease,dbDEMC
3	hsa-mir-16	HMDD, dbDEMC	28	hsa-let-7e	HMDD,mir2disease,dbDEMC
4	hsa-mir-155	HMDD,mir2disease,dbDEMC	29	hsa-mir-205	HMDD,mir2disease,dbDEMC
5	hsa-mir-125b	HMDD,mir2disease,dbDEMC	30	hsa-let-7d	HMDD,mir2disease,dbDEMC
6	hsa-mir-17	HMDD, dbDEMC	31	hsa-let-7b	HMDD, dbDEMC
7	hsa-mir-34a	HMDD, dbDEMC	32	hsa-let-7i	HMDD,dbDEMC,miR2disease
8	hsa-mir-19a	HMDD, dbDEMC	33	hsa-let-7c	HMDD,dbDEMC
9	hsa-mir-15a	HMDD, dbDEMC	34	hsa-let-7f	HMDD,mir2disease,dbDEMC
0	hsa-mir-373	HMDD,mir2disease,dbDEMC	35	hsa-mir-9	HMDD,dbDEMC
11	hsa-mir-221	HMDD, miR2disease	36	hsa-let-7g	HMDD,dbDEMC
12	hsa-mir-20a	HMDD, dbDEMC	37	hsa-mir-145	HMDD,mir2disease,dbDEMC
13	hsa-mir-451	HMDD, miR2disease	38	hsa-mir-146b	HMDD, miR2disease
14	hsa-mir-18a	HMDD, dbDEMC	39	hsa-mir-143	HMDD,mir2disease,dbDEMC
15	hsa-mir-29c	HMDD, dbDEMC	40	hsa-mir-181a	HMDD,dbDEMC, miR2Disease
16	hsa-mir-29a	HMDD, dbDEMC	41	hsa-mir-92b	dbDEMC
17	hsa-mir-19b	HMDD, dbDEMC	42	hsa-mir-127	HMDD,mir2disease,dbDEMC
18	hsa-mir-222	HMDD, dbDEMC	43	hsa-mir-29b	HMDD,mir2disease,dbDEMC
19	hsa-mir-302b	HMDD, miR2disease	44	hsa-mir-137	HMDD,dbDEMC
20	hsa-mir-92a	HMDD, dbDEMC	45	hsa-mir-126	HMDD,mir2disease,dbDEMC
21	hsa-mir-181b	HMDD,mir2disease,dbDEMC	46	hsa-mir-184	Unconfirmed
22	hsa-let-7a	HMDD,mir2disease,dbDEMC	47	hsa-mir-15b	dbDEMC
23	hsa-mir-372	HMDD, dbDEMC	48	hsa-mir-101	HMDD,dbDEMC,miR2disease
24	hsa-mir-200b	HMDD,mir2disease,dbDEMC	49	hsa-mir-200a	HMDD,mir2disease,dbDEMC
25	hsa-mir-223	HMDD, dbDEMC	50	hsa-mir-150	HMDD, dbDEMC

Forty-nine of the top 50 potential breast cancer miRNAs candidates have been confirmed based on the update HMDD, dbDEMC and mir2disease.

**Table 4:** The top 50 colon cancer-related miRNAs candidates predicted by GSTRW with removed all known colon cancer-miRNA associations and the confirmation of these associations.

Rank	miRNA name	evidences	Rank	miRNA name	evidences
1	hsa-mir-21	HMDD,miR2Disease,dbDEMC	26	hsa-mir-10a	dbDEMC,miR2Disease
2	hsa-mir-15a	HMDD,dbDEMC	27	hsa-mir-141	HMDD,miR2Disease,dbDEMC
3	hsa-mir-16	HMDD,dbDEMC	28	hsa-let-7d	HMDD,dbDEMC
4	hsa-mir-155	HMDD,miR2Disease,dbDEMC	29	hsa-mir-205	HMDD,dbDEMC
5	hsa-mir-17	HMDD,dbDEMC	30	hsa-let-7b	HMDD,miR2Disease,dbDEMC
6	hsa-mir-34a	HMDD,miR2Disease,dbDEMC	31	hsa-let-7i	HMDD,dbDEMC
7	hsa-mir-451	dbDEMC,miR2Disease	32	hsa-mir-145	HMDD,miR2Disease,dbDEMC
8	hsa-mir-19a	HMDD,miR2Disease,dbDEMC	33	hsa-let-7f	HMDD,dbDEMC
9	hsa-mir-125b	dbDEMC	34	hsa-mir-223	HMDD,miR2Disease,dbDEMC
10	hsa-mir-373	Unconfirmed	35	hsa-let-7e	HMDD,dbDEMC
11	hsa-mir-221	HMDD,miR2Disease,dbDEMC	36	hsa-let-7c	HMDD,dbDEMC
12	hsa-mir-20a	HMDD,miR2Disease,dbDEMC	37	hsa-mir-9	dbDEMC
13	hsa-mir-146a	HMDD,dbDEMC	38	hsa-let-7g	HMDD,miR2Disease,dbDEMC
14	hsa-mir-18a	HMDD,miR2Disease,dbDEMC	39	hsa-mir-181a	dbDEMC,miR2Disease
15	hsa-mir-29c	dbDEMC	40	hsa-mir-137	HMDD,dbDEMC,miR2Disease
16	hsa-mir-29a	HMDD,dbDEMC,miR2Disease	41	hsa-mir-92b	Unconfirmed
17	hsa-mir-222	dbDEMC	42	hsa-mir-127	HMDD,miR2Disease,dbDEMC
18	hsa-mir-181b	dbDEMC,miR2Disease	43	hsa-mir-126	HMDD,dbDEMC
19	hsa-mir-19b	HMDD,miR2Disease,dbDEMC	44	hsa-mir-29b	HMDD,miR2Disease,dbDEMC
20	hsa-mir-302b	HMDD,dbDEMC	45	hsa-mir-146b	dbDEMC
21	hsa-mir-92a	HMDD,dbDEMC	46	hsa-mir-199a	pubmed: 20226080
22	hsa-let-7a	HMDD,miR2Disease,dbDEMC	47	hsa-mir-15b	dbDEMC,miR2Disease
23	hsa-mir-372	dbDEMC,miR2Disease	48	hsa-mir-200a	Unconfirmed
24	hsa-mir-143	HMDD,miR2Disease,dbDEMC	49	hsa-mir-122	dbDEMC
25	hsa-mir-200b	HMDD,dbDEMC	50	hsa-mir-196a	dbDEMC,miR2Disease

Forty-six of the top 50 potential conlon cancer miRNAs candidates have been confirmed based on the update HMDD, dbDEMC and mir2disease.

For colon cancer, the association of 37 known miRNAs with colon cancer was removed. Of the first 50 miRNAs predicted by GSTRW, 46 were validated in the above three databases, and four were unidentified are hsa-mir-373, hsa-mir-92b, hsa-mir-199a and hsa-mir-200a, all of which are predicted in previous colon cancer examples.Therefore, we believe that GSTRW performs well in predicting the performance of isolated diseases.

All data sets used in this paper are generated before the literature is published. Therefore, it further illustrates the reliable performance of the proposed method in this paper.

## Discussions

MiRNA is closely related to diseases. More scholars are exploring the use of miRNA in the diagnosis, classification and treatment of diseases. The effective computation method that can be used to identify miRNA–disease association can contribute to experimental studies on miRNA. In this paper, a miRNA–disease association prediction algorithm based on the two-tier global similarity (GSTRW) is proposed to predict miRNA–disease association. On the basis of the miRNA–miRNA similarity, miRNA family information and disease similarity, we use the Laplacian score of graphs to calculate the global similarity of miRNA and disease. miRNA association information of the similar disease (miRNA) is introduced to optimise disease seed nodes. Then, they randomly walk in the miRNA global similarity network and the disease global similarity network, respectively. After obtaining two stable distributions, we use the Pearson correlation to calculate miRNA–disease association prediction scores. Finally, the two scores are weighted to obtain the final miRNA–disease association score. A cross validation and a case study reveal that GSTRW is a type of global method that can predict the association between all diseases and miRNA compared with those of the most advanced computation method. Moreover, it can be utilised to predict the isolated diseases and new miRNA, and negative samples are not needed.

The excellent performance of GSTRW is mainly attributed to the following factors. Firstly, our algorithm integrates many biological information, including miRNA functional similarity, miRNA family information, disease similarity and miRNA–disease information, to establish the global similarity network by combining with the Laplacian score of graphs. Therefore, the prediction performance is improved. Secondly, the random walk algorithm refers to walking in the miRNA global and disease global similarity networks. Therefore, it fully considers the global similarity of miRNAs and diseases and optimises the initial walking operator.

GSTRW is a valuable computing tool that can be used to predict the association of disease and disease. This method can be further applied to reveal other biological associations, such as lncRNA–disease, gene–disease and drug–target associations. Our method has achieved good results, but it also has some limitations. Firstly, our method has more parameters. The mechanism of quickly and simply determining the parameters in GSTRW has yet to be investigated. Secondly, a reasonable approach to build miRNA similarity and disease similarity can help improve our predictive performance. More importantly, the cancer hallmarks^82,83^ is really helpful for predicting tumor clinical phenotypes. In future study, we will do further analysis between miRNAs and cancer hallmarks.We plan to integrate more biological information such as cancer hallmark and define miRNA and disease similarities.

## Methods

### Dataset and preprocessing

Two data sets are used in this study. A total of 270 miRNA–disease association pairs are obatained from ref.^19^, and 19 miRNAs that cannot be found in a previous study^35^ are removed. Finally, 99 miRNAs and 51 diseases, including 225 miRNA–disease pairs, are retained. This data set is called gold benchmark data set. Another miRNA–disease association data set is obatained from ref.^35^ to validate the insensitivity of our method to the data set. This data set includes 1616 human miRNA–disease associations verified by the experiments. After integrating different miRNA records and unifying the miRNA and disease names, we finally reserve 1395 miRNA–disease associations, including 271 miRNAs and 137 diseases. This data set is named predictive dataset.

MiRNA–miRNA functional similarity score is obtained from a previous study^35^, and this data set has been successfully applied to many methods^21,42–44^. Matrix SM is used to represent the adjacency matrix of miRNA, and SM (i, j) refers to the functional similarity score between miRNA i and miRNA j.

Disease similarity data are obtained from another study^84^. Matrix SD is used to represent the adjacency matrix of disease, and SD (i, j) refers to the functional similarity score between diseases i and j.

MiRNA family information is obtained from the miRBase database^85^. Studies have shown that miRNAs in the same family have more mRNA targets than those of miRNAs in different families, thereby indicating a higher functional similarity in the former than in the latter^34^. Matrix SM^fam^ is used to represent miRNA family information. If two miRNAs are in the same family, then SMfam (i, j) is set to 1; otherwise, SM^fam^ is 0.

### miRNA and disease similarity networks

We integrate the functional similarity score and family information of miRNA to build an miRNA similarity network:

1 $$SIM(i,j)=SM(i,j)\times (1+S{M}^{fam}(i,j))$$

where SIM (i, j) refers to the similarity score between miRNAs i and j after information fusion is performed, SM (i, j) indicates the similarity score between miRNAs i and j, and SMfam corresponds to the miRNA family information matrix. When miRNA i and miRNA j belong to the same family, SM^fam^ (i, j) is equal to 1. The similarity score of two miRNAs is twice the function score, indicating that miRNAs have a high similarity.

A disease similarity network is built by directly using the phenotypic information of diseases^84^. Phenotypic similarity after data processing can be represented by matrix SD. The node in the disease similarity network corresponds to the disease in SD, and the similarity between diseases is represented by the edge between the corresponding nodes with weight. If the weight of the edge is high, then the corresponding diseases are highly similar.

### Global similarity calculation based on the Laplacian score of graphs

Laplacian score of graphs has been successfully applied^42,43,86^. In the global similarity of a particular disease to be inquired with other diseases in a given network, the global association of one miRNA with other miRNAs in the network is obtained by calculating the Laplacian score of graphs.

In this study, the binary vector d = {d_1_, d_2_, …, d_n_} is used to represent the initial vector of the disease to be inquired (d_i_). The corresponding element value of d_i_ is 1, and other elements are 0. The global similarity between d_i_ and other diseases is obtained by calculating the Laplacian score of graphs represented by $\mathop{d}\limits^{ \sim }$, which can be obtained by solving following optimisation equation^87^:

2 $${{\rm{\min }}}_{\alpha }\sum _{i,j}{\overline{SD}}_{i,j}{({\tilde{d}}_{i}-{\tilde{d}}_{j})}^{2}+\frac{1-\alpha }{\alpha }\sum _{i}{({\tilde{d}}_{i}-{\tilde{d}}_{j})}^{2}$$

In Eq. (2), the first item is a smooth penalty item, and $\overline{SD}$ is the column normalization matrix of matrix SD. With this parameter, a similar score for the related diseases can be obtained. The second item ensures the consistency of the disease to be inquired with other diseases, and α is a balance factor, where α ∈ (0, 1). It is used to balance the two penalty items in Eq. (2). The approximate solution of Eq. (2) is as follows^87^:

3 $$\tilde{d}=(1-\alpha ){(I-\alpha \overline{SD})}^{-1}d$$

Using this method, we can obtain the global similarity scores among all of the diseases in all of the disease networks as represented by matrix $sim\tilde{D}$.

Using a similar method, we can obtain the similarity between the inquired miRNA mj and other miRNAs:

4 $$\tilde{m}=(1-\beta ){(I-\beta \overline{SIM})}^{-1}m$$

where $\overline{SIM}$ is the column normalization matrix of matrix SIM, *β* is the balance factor, and $\beta \in (0,1)$. The global similarity matrix of all miRNAs in the miRNA network is recorded as $sim\tilde{M}$.

### Calculation method for the global similarity score of the miRNA–disease association based on the two-tier network random walk

On the basis of our hypothesis that miRNA with functional similarity is usually associated with a disease exhibiting a phenotypic similarity, we design a Global Similarity method based on a Two-tier network Random Walk for the prediction of disease association (GSTRW) to reveal the association between a novel miRNA and a disease. We aim to include the following: (1) the known miRNA–disease information, (2) the global similarity between a particular disease and other diseases, (3) the global similarity between a specific miRNA and other miRNAs and (4) information regarding the miRNA family.

**Figure 7:**
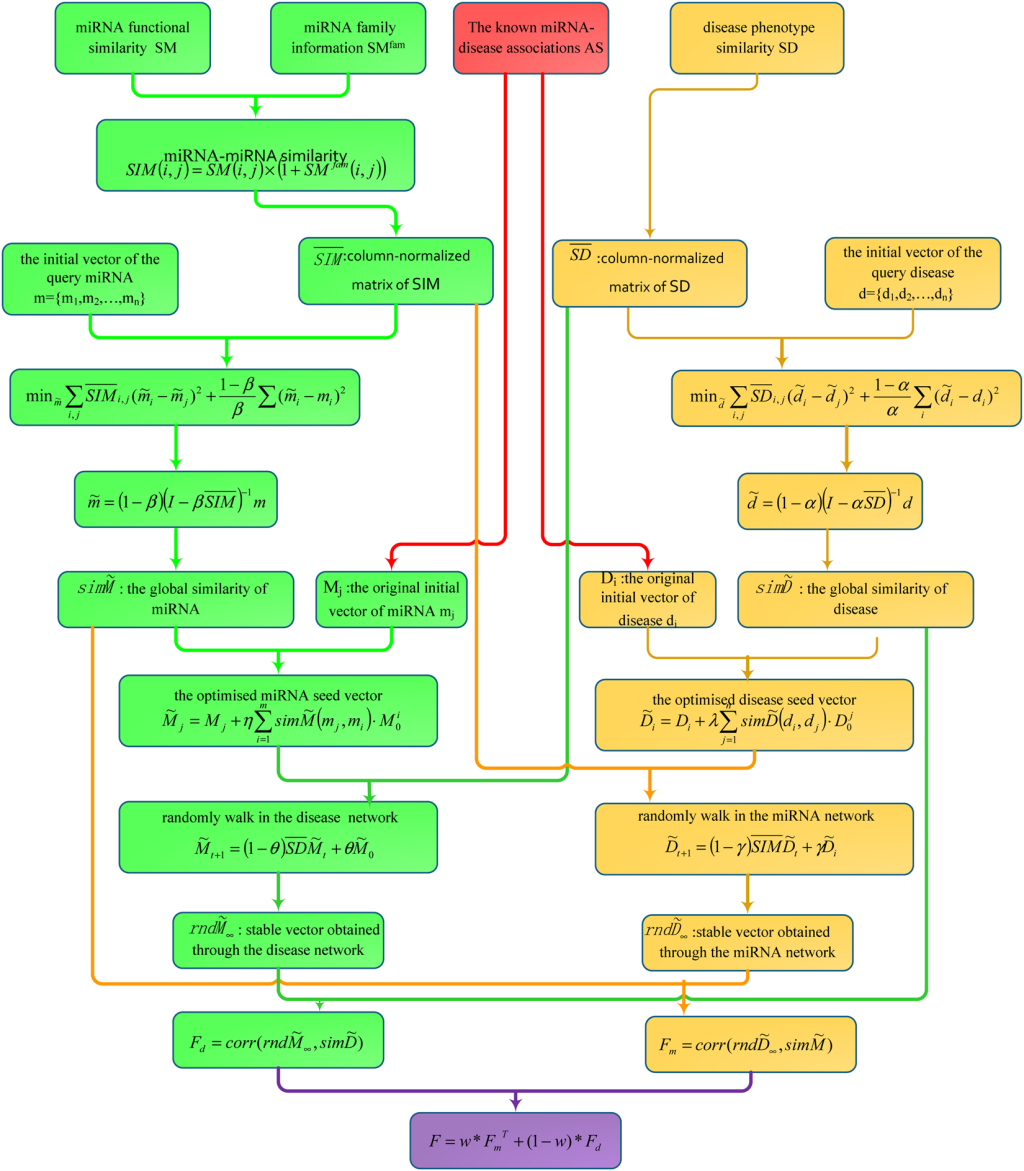
The overall flowchart of GSTRW.

Firstly, we instruct the optimised disease seed to walk in the miRNA network and thus obtain a stable vector. The Pearson coefficient of this stable vector and the global similarity between the inquired miRNA mj calculated using Eq. (4) and the other miRNAs are used as the predictive scores of the disease in the miRNA global similarity network. And then, we instruct the optimized miRNA seed to walk in the disease network and thus obtain a stable vector. The Pearson coefficient of this stable vector and the global similarity between the inquired disease d_i_ calculated using Eq. (3) and other diseases are used as the predictive scores of miRNA in the disease global similarity network. Finally, these two scores are weighted to obtain the final miRNA–disease association prediction score. If the score is high, then miRNA m_j_ likely causes d_i_. The specific flow chart is shown in Fig. 7, and the calculation is described below.

To carry out the random walk in the miRNA and disease similarity networks, we should firstly determine the seed sequence. To apply our algorithm to the association prediction of the isolated disease on the basis of our hypothesis, we introduce the miRNA-associated information of the similar disease and consequently solve the problems on the disease–miRNA association prediction, considering the completely unknown miRNA association information of the isolated diseases. Seed calculation formula isshown as below:

5 $${\tilde{D}}_{i}={D}_{i}+\lambda \sum _{j=1}^{n}sim\tilde{D}({d}_{i},{d}_{j})\cdot {D}_{0}^{j}$$

where ${\tilde{D}}_{i}$ refers to the initial vector of the optimised seed, and Di corresponds to the original initial vector of d_i_ to save the information of d_i_ in the initial stage associated with all miRNAs. If miRNA is correlated with d_i_, then the corresponding position is assigned as 1; otherwise, the corresponding position is 0. $sim\tilde{D}({d}_{i},{d}_{j})$ denotes the global similarity between d_i_ and d_j_, and their similarity can be obtained from the global correlation vector d~ of d_i_ calculated from Eq. (3). ${D}_{0}^{j}$ refers to the initial vector of d_j_, that is, the known miRNA-associated information of d_j_. n refers to the total number of diseases, while λ is the balance parameter. Therefore, miRNA information associated with a similar disease is introduced to optimise the initial associated miRNA of d_i_.

After the initial vector is obtained, the restarted random walk can be carried out in the miRNA similarity network to obtain a stable information distribution vector. The random walk formula is expressed as Eq. (6).

6 $${\tilde{D}}_{t+1}=(1-\gamma )\overline{SIM}{\tilde{D}}_{t}+\gamma {\tilde{D}}_{i}$$

where $\overline{SIM}$ refers to the column normalization matrix of the similar matrix SIM, *γ* refers to the probability of the restart, and $\gamma \in (0,1)$. ${\tilde{D}}_{t}$ represents the information distribution after *t* times of iteration. After several times of iteration, the probability space reaches a stable state: ${\tilde{D}}_{\infty }(|{\tilde{D}}_{t+1^{\prime} }-{\tilde{D}}_{t^{\prime} }| < {10}^{-6})$. Thus, the iteration can be stopped. The walk results of all diseases in the miRNA similarity network are represented by matrix $rnd{\tilde{D}}_{\infty }$.

After obtaining the distribution vector, we use the Pearson coefficient of the distribution vector to determine the predictive score of the disease for the disease–miRNA association in the miRNA similarity network, which is represented as follows:

7 $${F}_{m}=corr(rnd{\tilde{D}}_{\infty },sim\tilde{M})$$

We instruct the optimised miRNA seed vector to randomly walk in the disease similarity network. The initial seed of miRNA mj is calculated as follows:

8 $${\tilde{M}}_{j}={M}_{j}+\eta \sum _{i=1}^{m}sim\tilde{M}({m}_{j},{m}_{i})\cdot {M}_{0}^{i}$$

where ${\tilde{M}}_{j}$ refers to the obtained initial vector of seed, and *M*_*j*_ corresponds to the original initial vector of miRNA m_j_ to save the miRNA mj-associated information with other diseases in the initial state. If the disease is associated with miRNA m_j_, then the corresponding position is assigned as 1; otherwise, it is 0. $sim\tilde{M}({m}_{j},{m}_{i})$ denotes the global similarity between miRNA m_j_ and miRNA m_i_. ${M}_{0}^{i}$ is the initial vector of miRNAi, that is, the known miRNA m_i_–disease association information. *m* refers to the total number of miRNAs, and η is the balance parameter. After obtaining the initial vector, we perform the restarted random walk in the disease similarity network. Eq. (9) is expressed as follows:

9 $${\tilde{M}}_{t+1}=(1-\theta )\overline{SD}{\tilde{M}}_{t}+\theta {\tilde{M}}_{0}$$

where $\overline{SD}$ refers to a column normalization matrix of the similarity matrix SD, and *θ* corresponds to the probability of the restart, $\theta \in (0,1)$. After several times of iteration, the probability space reaches a stable state: ${\tilde{M}}_{\infty }(|{\tilde{M}}_{t+1^{\prime} }-{\tilde{M}}_{t^{\prime} }| < {10}^{-6})$; thus, the iteration can be stopped. The walking result of all miRNAs in the disease similarity network is represented by matrix $rnd{\tilde{M}}_{\infty }$.

After obtaining the distribution vector, we use the Pearson coefficient of the distribution vector to determine the predictive score of miRNA for the miRNA–disease association in the disease global similarity network.

10 $${F}_{d}=corr(rnd{\tilde{M}}_{\infty },sim\tilde{D})$$

Finally, the predictive score of disease in the miRNA global similarity network and the predictive score of miRNA in the disease global similarity network are weighted to obtain the final miRNA–disease association prediction score by using the following equation:

11 $$F=w\ast {{F}_{m}}^{T}+(1-w)\ast {F}_{d}$$

where Row i Column j in matrix F *F*(*i*, *j*) refers to the association score of miRNA i and disease j. If the score is high, then the degree of association is high.
